# The Association Between the Incidence Risk of Peripheral Neuropathy and PD-1/PD-L1 Inhibitors in the Treatment for Solid Tumor Patients: A Systematic Review and Meta-Analysis

**DOI:** 10.3389/fonc.2019.00866

**Published:** 2019-09-04

**Authors:** Zhihua Si, Shuisheng Zhang, Xiaowei Yang, Nan Ding, Meiyi Xiang, Qingshan Zhu, Yantao Mao, Yajuan Lv, Lili Yu, Heli Shang, Jian Xie, Yuan Tian

**Affiliations:** ^1^Department of Neurology, Shandong Provincial Qianfoshan Hospital, The First Hospital Affiliated with Shandong First Medical University, Jinan, China; ^2^Department of General Surgery, Peking University Third Hospital, Beijing, China; ^3^Department of Hepatobiliary Intervention, School of Clinical Medicine, Beijing Tsinghua Changgung Hospital, Tsinghua University, Beijing, China; ^4^Key Laboratory of Carcinogenesis and Translational Research (Ministry of Education/Beijing), Division of Etiology, Peking University Cancer Hospital and Institute, Beijing, China; ^5^Department of Radiotherapy Oncology, Anyang Tumor Hospital, Anyang, China; ^6^Department of Oncology, Yantaishan Hospital, Yantai, China; ^7^Department of Radiotherapy Oncology, Shandong Provincial Qianfoshan Hospital, The First Hospital Affiliated with Shandong First Medical University, Jinan, China

**Keywords:** incidence risk, peripheral neuropathy, PD-1/PD-L1, solid tumor, meta-analysis

## Abstract

**Purpose:** We conducted this study to determine the relationship between PD-1/PD-L1 inhibitors and the incidence risk of peripheral neuropathy in patients with solid tumors.

**Method:** The process of the meta-analysis was performed by us according to the Preferred Reporting Items for Systematic Reviews and Meta-analyses (PRISMA) guidelines. Incidence of all-grade and grade 3–5 treatment-related peripheral neuropathy in patients with solid tumors were taken into account.

**Results:** After screening and eligibility assessment, a total of 17 clinical trials involving 10,500 patients were selected for the final meta-analysis. The incidence risk of peripheral neuropathy for all grade was significantly lower in the PD-1/PD-L1 inhibitor group than that of the control group, either monotherapy (OR = 0.08, 95%CI:[0.03, 0.19]) or chemotherapy (OR = 0.05, 95%CI:[0.03, 0.11]). Similar incidence trend could also be seen for the incidence risk of grade 3–5 peripheral neuropathy. When PD-1/PD-L1 inhibitors were used in combination with chemotherapy, the incidence risk of peripheral neuropathy was higher than in the control chemotherapy group, whether it was all-grade (OR = 1.22, 95%CI:[1.00, 1.49]) or grade 3–5 degree (OR = 1.74, 95%CI:[1.03, 2.92]).

**Conclusion:** Compared with chemotherapy, incidence risk of peripheral neuropathy related to PD-1/PD-L1 inhibitor was significantly lower than that of the chemotherapy group, while PD-1/PD-L1 inhibitor increased the incidence risk of peripheral neuropathy when it was combined with chemotherapy.

## Introduction

Peripheral neuropathy is a syndrome characterized by loss of sensation, muscle weakness and atrophy, loss of tendon reflexes, and/or abnormal vascular motion as a clinical manifestation, either alone or in any combination. Drugs, especially for anti-tumor drugs, are one of the common pathogenic factors for the disease (1–10). During the course of anti-tumor therapy, whether it is chemotherapy or targeted therapy drugs (1–5), peripheral neuropathy is often reported as a drug side effect (1–10). Although reports of death due to peripheral neuropathy were rare, it seriously affected the quality of life for patients with malignant tumors (8–10). Therefore, peripheral neuropathy caused by anti-tumor drugs had increasingly attracted the attention of clinical doctors (11–13).

As a new targeted anti-tumor drug, PD-1/PD-L1 inhibitors have achieved satisfactory clinical efficacy for the treatment of solid tumors, either alone or in combination (14–29). With the increasing clinical applications, more and more drug-related side toxicity effects had been reported, and peripheral neuropathy was one of them (14–29). Because of the low incidence of peripheral neuropathy, we were unable to determine the association between its incidence risk and PD-1/PD-L1 inhibitors. Some chemotherapeutic drugs, such as paclitaxel, might cause delayed peripheral neuropathy (12, 13). It was impossible for us to identify the association between the incidence risk of peripheral neuropathy and PD-1/PD-L1 inhibitors when they were used in combination with other anti-tumor drugs or prescribed as a second-line treatment after chemotherapy (14–30).

For drug-induced peripheral neuropathy, stopping the drug remained to be the primary treatment method (1–10). However, for patients with malignant tumors, when severe drug side effects were encountered (12, 13), careful consideration for stopping the drug should be taken into account. Because of the sudden stop of anti-tumor treatment, it was very likely to cause rapid progression of the tumor. When PD-1/PD-L1 inhibitors were used in combination with chemotherapy, it was particularly important to determine the cause of peripheral neuropathy and then decide which drug to be discontinued (15–18).

To solve the above problems and clarify the association between incidence risk of peripheral neuropathy and PD-1/PD-L1 inhibitors, we designed this meta-analysis.

## Methods

The process of the meta-analysis was performed according to the Preferred Reporting Items for Systematic Reviews and Meta-analyses (PRISMA) guidelines (31).

### Types of Enrolled Studies

According to the research design, the selected clinical studies must meet the following criteria: (1) Randomized controlled clinical trials would be prioritized, (2) PD-1/PD-L1 inhibitor was prescribed for at least one group of participants, (3)The control group was an anti-tumor drug or PD-1/PD-L1 in combination with an anti-tumor drug rather than a placebo, (4) Participants were diagnosed with solid malignant tumors rather than hematological malignancy, (5) Data on peripheral neuropathy were reported in the study, (6) the enrolled study was published in English.

### Search Strategy

Original articles including PD1/PD-L1 inhibitor regimens for solid tumor patients were verified by a systematic search of PubMed. The reported date of the results was limited from Jan 22, 2013 to May 31, 2019. The following subject terms would be used in the literature search process: “cancer,” “tumor,” “PD1/PD-L1,” “nivolumab,” “Opdivo,” “pembrolizumab,” “Keytruda,” “Imfinzi,”,“MK-3475,” “atezolizumab,” “Tecentriq,” “MPDL3280A,” “avelumab,” “Bavencio,” “durvalumab,” “BMS-963558.” Studies limited in human beings, shown in full text, abstract, or poster form, were selected three investigators (Shuisheng Zhang, Yi Zhao, Qingshan Zhu) were appointed to check eligibility and duplicate independently by screening titles and abstracts of relevant studies. If data on peripheral neuropathy had not been reported, we would contact the corresponding author of the article to verify it again, or it would be precluded from the meta-analysis. The basic characteristics information included in the study would be summarized in Table 1.

**Table 1:** Basic characteristics of the included studies.

**No**.	**Study name**	**Drug name**	**Drug type**	**Treatment regimen**	**Number of evaluable patients**	**Peripheral neuropathy**	**Previous therapy**	**Phase**	**Randomized controlled trial (RCT)**	**Tumor type**
1	Cohen et al. (14)	Pembrolizumab	PD-1	Pembrolizumab vs. (Methotrexate, Docetaxel, or Cetuximab)	480	7	Platinum-based	III	RCT	Head-and-neck squamous cell carcinoma
2	Schmid et al. (15)	Atezolizumab	PD-L1	Atezolizumab + Nab-paclitaxel vs. Placebo + Nab-paclitaxel	890	195	NO	III	RCT	Advanced Triple-Negative Breast Cancer (BC)
3	Horn et al. (16)	Atezolizumab	PD-L1	Atezolizumab + Carboplatin + Etoposide vs. Placebo + Carboplatin + Etoposide	394	6	NO	III	RCT	SCLC
4	Socinski et al. (17)	Atezolizumab	PD-L1	Atezolizumab + BCP vs. BCP	787	274	NO	III	RCT	Metastatic non squamous NSCLC
5	Paz-Ares et al. (18)	Pembrolizumab	PD-1	Pembrolizumab + Carboplatin + Paclitaxel vs. Placebo + Carboplatin + Paclitaxel	558	102	NO	III	RCT	Metastatic squamous NSCLC
6	Shitara et al. (19)	Pembrolizumab	PD-1	Pembrolizumab vs. Paclitaxel	570	41	YES	III	RCT	Advanced gastric or gastro-oesophageal junction cancer
7	Powles et al. (20)	Atezolizumab	PD-L1	Atezolizumab vs. Chemotherapy	1,128	53	Platinum-based	III	RCT	Locally advanced or metastatic urothelial carcinoma (UC)
8	Hida et al. (21)	Atezolizumab	PD-L1	Atezolizumab vs. Docetaxel	101	14	Platinum-based	III	RCT	Locally advanced/metastatic NSCLC
9	Bellmunt et al. (22)	Pembrolizumab	PD-1	Pembrolizumab vs. Chemotherapy	521	28	Platinum-based	III	RCT	Advanced Urothelial Carcinoma (UC)
10	Rittmeyer et al. (23)	Atezolizumab	PD-L1	Atezolizumab vs. Docetaxel	1187	89	Platinum based	III	RCT	Squamous or non squamous NSCLC
11	Ferris et al. (24)	Nivolumab	PD-1	Nivolumab vs. (Methotrexate, Docetaxel, or Cetuximab)	347	8	Platinum-based	III	RCT	Recurrent Squamous-Cell Carcinoma of the Head and Neck
12	Antonia et al. (25)	Nivolumab	PD-1	Nivolumab vs. Nivolumab + Ipilimumab	213	1	Platinum-based	I/II	N/A	Recurrent SCLC
13	Fehrenbacher et al. (26)	Atezolizumab	PD-L1	Atezolizumab vs. Doctaxel	277	16	Platinum-based	II	RCT	NSCLC
14	Herbst et al. (27)	Pembrolizumab	PD-1	Pembrolizumab vs. Docetaxel	991	33	Platinum-containing	II/III	RCT	Advanced NSCLC
15	Borghaei et al. (28)	Nivolumab	PD-1	Nivolumab vs. Docetaxel	555	28	Platinum-based	III	RCT	Non-squamous NSCLC
16	Brahmer et al. (29)	Nivolumab	PD-1	Nivolumab vs. Docetaxel	260	16	Platinum-based	III	RCT	Squamous NSCLC
17	Mok et al. (30)	Pembrolizumab	PD-1	Pembrolizumab vs. Chemotherapy	1,241	51	NO	III	RCT	NSCLC

*RCT, Randomized controlled trial; BCP, Bevacizumab plus Carboplatin plus Paclitaxel; NSCLC, Non-Small Cell Lung Cancer; PD-1, Programmed Cell Death 1; PD-L1, Programmed Cell Death Ligand 1; SCLC, Small Cell Lung Cancer; CP, Carboplatin plus Paclitaxel; PC, Pemetrexed plus a platinum-based drug; Chemotherapy, Carboplatin plus Pemetrexed, Cisplatin plus Pemetrexed, Carboplatin plus Gemcitabine, Cisplatin plus Gemcitabine, or Carboplatin plus Paclitaxel; N/A, No Available*.

### Assessment of Study Quality and Publication Bias

Funnel plot, Egger's test and Newcastle-Ottawa scale, proposed by the Cochrane Collaboration, were taken to evaluate the bias (31–35). Three investigators (Shuisheng Zhang, Yi Zhao, Qingshan Zhu) were appointed to check the quality of all studies. The results, including random sequence generation, allocation concealment, blinding of participants and personnel, blinding of outcome assessment, incomplete outcome data, and selective outcome reporting, would be summarized in a figure together.

### Outcome and Exposure of Interest

The study name, year, phase, tumor type, PD-1 and PD-L1 inhibitor regimen, previous therapy regimen, number of evaluable cases, and number of peripheral neuropathy events were extracted from every enrolled study. Both all-grade and grade 3–5 peripheral neuropathy data were taken into account for the final comprehensive meta-analysis.

### Assessment of Heterogeneity and Statistical Analysis

Cochrane's Q statistic and the *I*^2^ statistic were taken into account for evaluating the heterogeneity among enrolled studies just as suggested by Moher et al. (31) and Higgins et al. (36). The grade of heterogeneity was calculated by the range of *I*^2^ values. Heterogeneity was considered low, moderate or high according to *I*^2^ values <25%, 25–50%, and >50%, respectively.

Odds ratio (OR) value was reported to be a much more conservative evaluation parameter and might be more inclined to reveal a safety signal, as the method by which an OR is calculated provided a point estimate farther from unity than that provided by a HR. Odds ratio (OR), and 95% confidence interval (CI) would be calculated by random effect (RE) (37). Risk Ratio (RR) and Risk Difference (RD) were also calculated as secondary reference indicators for a more detailed interpretation of the results. *P* < 0.05 was considered to be of statistically significance. In order to clarify the correlation between peripheral neuropathy and PD-1/PD-L1 inhibitors, we performed a large number of subgroup analyses based on the type of tumor, the treatment regimen and the specific drug. The software of Review Manager 5.3 was used for data consolidation and analysis. Statistical tests were all two-sided.

## Results

### Literature Search Results

According to the searching principle set by our team, 505 related documents were retrieved on the PubMed website, and 58 related documents were found in other websites or published documents.

**Figure 1:**
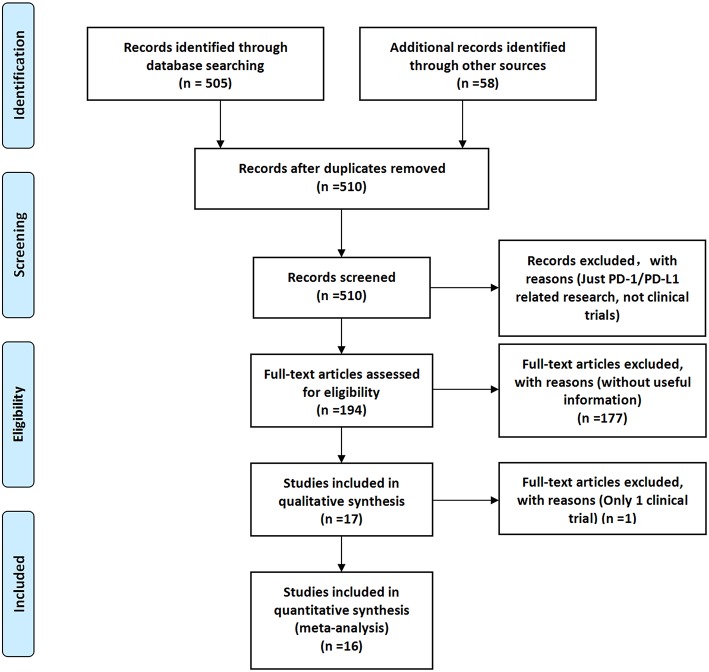
Study flow diagram of inclusion.

After screening and eligibility assessment, a total of 17 clinical trials involving 10,500 patients were selected for the final meta-analysis. The flow diagram of the meta-analysis was shown in Figure 1, while the risk of bias summary was shown in Supplemental Figure 1. All clinical trials enrolled in the meta-analysis included at least one experimental group and one control group (14–30).

### Characteristics of Identified Trials

The basic characteristics of all the enrolled clinical trials were summarized in Table 1 (14–30). The involving PD-1/PD-L1 inhibitors were nivolumab (*n* = 4) (24, 25, 28, 29), pembrolizumab (*n* = 6) (14, 18, 19, 22, 27, 30), and atezolizumab (*n* = 7) (15–17, 20, 21, 23, 26). Of all the clinical trials included, 14 were phase III (14–24, 28–30), 1 was phase II (26), 1 was phase II/III (27), and 1 was phase I/II (25). The tumors involved in 17 clinical trials included lung cancer (*n* = 11) (16–18, 21, 23, 25–30), urothelial cancer (*n* = 2) (20, 22), breast cancer (*n* = 1) (15), head and neck carcinoma (*n* = 2) (14, 24), and advanced gastric or gastro-esophageal junction cancer (*n* = 1) (19). Of the 11 lung cancer-related clinical trials, nine were limited to non-small cell lung cancer (NSCLC) and two were limited to small cell lung cancer (SCLC) (16, 25). 16 clinical trials were reported to be randomized controlled trial (RCT) (14–24, 26–30), while the information of one clinical trial was unavailable (25). Twelve trials underwent previous platinum-based treatments before PD-1/PD-L1 inhibitors (14, 19–29), while PD-1/PD-L1 inhibitors were prescribed as the first line therapy regimens for the other five trials (15–18, 30). The drugs used in 10 clinical trials were PD-1 inhibitors (14, 18, 19, 22, 24, 25, 28–30), while PD-L1 inhibitors were just given for the other seven clinical trials (15–17, 20, 21, 23, 26).

### Risk of Bias

Study quality and risk of bias among enrolled studies were checked by Newcastle-Ottawa scale (35). Random sequence generation (selection bias), allocation concealment (selection bias), blinding of participants and personnel (performance bias), blinding of outcome assessment (detection bias), incomplete outcome data (attrition bias), and selective reporting (reporting bias) were assessed by three members of our team independently and summarized in Supplemental Figure 1. Publication bias, evaluated by Harbord's test (31), was displayed by funnel plots (Supplemental Figures 2, 3, 5, 7, 9, 11).

### Incidence Risk of All-Grade Peripheral Neuropathy

All included clinical trials were divided into four groups according to different treatment options, and the specific groups were as follows: Group A (PD-1/PD-L1 vs. Mono-therapy) (19, 21, 23, 26–29), Group B (PD-1/PD-L1 vs. Chemotherapy) (14, 20, 22, 24, 30), Group C (PD-1/PD-L1+ Chemotherapy vs. Chemotherapy) (15–18), Group D (PD-1 vs. PD-1+ CTLA-4) (25). Each group was further divided into two subgroups depending on the respective specific drug and tumor type. Meta-analysis was not performed in group D, because only one group of clinical trials was enrolled, and only one patient in both subgroups was reported with peripheral neuropathy (25).

**Figure 2:**
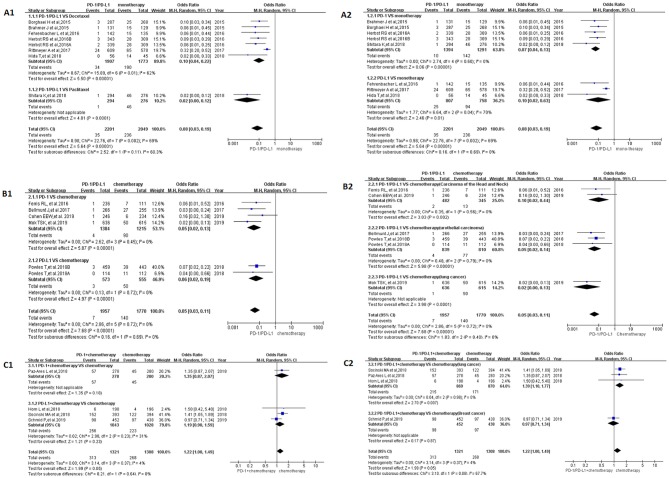
Forest plots for the odds ratio of treatment related peripheral neuropathy for all grade. **(A1)** Forest plots for the odds ratio of treatment related peripheral neuropathy (PD-1/PD-L1 vs. Docetaxel/Paclitaxel). Subgroup analysis was performed according to the type of chemotherapy drug in the control group. **(A2)** Forest plots for the odds ratio of treatment related peripheral neuropathy (PD-1/PD-L1 vs. monotherapy). Subgroup analysis was performed based on the drug type (PD-1 or PD-L1) of the experimental group. **(B1)** Forest plots for the odds ratio of treatment related peripheral neuropathy (PD-1/PD-L1 vs. Chemotherapy). Subgroup analysis was performed based on the drug type (PD-1 or PD-L1) of the experimental group. **(B2)** Forest plots for the odds ratio of treatment related peripheral neuropathy (PD-1/PD-L1 vs. Chemotherapy). Subgroup analysis was performed based on the specific types of tumors in the experimental and control groups. **(C1)** Forest plots for the odds ratio of treatment related peripheral neuropathy (PD-1/PD-L1 + Chemotherapy vs. Chemotherapy). Subgroup analysis was performed based on the drug type (PD-1 or PD-L1) of the experimental group. **(C2)** Forest plots for the odds ratio of treatment related peripheral neuropathy (PD-1/PD-L1 + Chemotherapy vs. Chemotherapy). Subgroup analysis was performed based on the specific types of tumors in the experimental and control groups.

We first performed a meta-analysis on the data of Group A, and the results of the analysis were summarized at the bottom of Figure 2A [OR = 0.08, 95%CI:[0.03, 0.19], I^2^ = 69%, Z = 5.64 (*P* < 0.00001)] (19, 21, 23, 26–29). Subgroup analysis was performed according to the different drug types in the control group and the experimental group, and the results were shown in Figures 2A1,A2, respectively. Moderate heterogeneity was found in Group A (*I*^2^ = 69%). Subgroup analysis results suggested that the source of heterogeneity was the PD-L1 subgroup [Figure 2A2; (21, 23, 26)]. The funnel plots of OR for Group A could be seen in Supplemental Figures 2A1,A2. Similar to the results of OR, RR and RD of Group A were displayed in Supplemental Figures 4A, 6A.The corresponding funnel plots were gathered in Supplemental Figures 5A, 7A.

When PD-1/PD-L1 drugs were compared with chemotherapy (Group B), the incidence of peripheral neuropathy was significantly lower than that of the control group, and the OR results are summarized in Figure 2B [OR = 0.05, 95%CI:[0.03, 0.11], I^2^ = 0%, Z = 7.68 (*P* < 0.00001)] (14, 20, 22, 24, 30). The funnel plots of OR for Group B could be seen in Supplemental Figures 2B1,B2. The subgroup analysis results were also similar to the subgroup analysis results of group A. RR and RD of Group B were displayed in Supplemental Figures 4B, 6B.The corresponding funnel plots were gathered in Supplemental Figures 5B, 7B. No obvious heterogeneity was found among Group B (*I*^2^ = 0%).

Different from the met-analysis results of group A and group B, we found that the analysis results of OR were not statistically significant when performing meta-analysis on Group C (Figure 2C) [OR = 1.22, 95%CI:[1.00, 1.49], *I*^2^ = 4%, *Z* = 1.99 (*P* = 0.05)] (15–18). The same trend could be seen in the results of RD (Supplemental Figure 6C) [RD = 0.03, 95%CI:[0.01, 0.06], *I*^2^ = 47%, *Z* = 1.42(*P* = 0.16)] (15–18). The corresponding funnel plots of them were gathered in Supplemental Figures 2C, 7C. The RR of Group C showed that the incidence risk of peripheral neuropathy in the PD-1/PD-L1 combined chemotherapy subgroup was significantly higher than that in the chemotherapy subgroup, and the *P*-value was statistically significant (Supplemental Figure 4C) [RR = 1.16, 95%CI:[1.01, 1.34], *I*^2^ = 0%, *Z* = 2.13(*P* = 0.03)] (15–18). The corresponding funnel plots of RR were gathered in Supplemental Figure 5C. No obvious heterogeneity was found among Group C (*I*^2^ = 0%).

### Incidence Risk of Grade 3–5 Peripheral Neuropathy

Twelve clinical trials with the information of grade 3–5 peripheral neuropathy were taken into account for further meta-analysis (15–20, 22, 23, 27–30). The same grouping and subgroup approach as before were taken for dealing with them. In the experimental subgroup of Group A and Group B, using PD-1/PD-L1 inhibitors alone, the incidence rate of peripheral neuropathy was 0% (19, 20, 22, 23, 27–30). In other words, in patients with solid tumors treated with PD-1/PD-L1 alone, the incidence rate of grade 3–5 peripheral neuropathy was 0% (19, 20, 22, 23, 25, 27–30).

**Figure 3:**
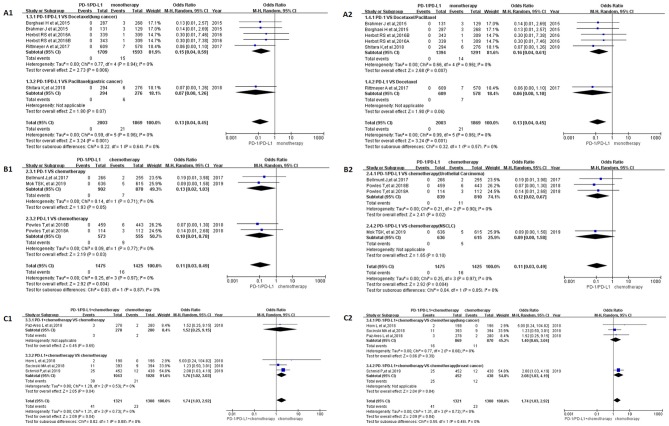
Forest plots for the odds ratio of treatment related peripheral neuropathy for grade 3–5. **(A1)** Forest plots for the odds ratio of treatment related peripheral neuropathy (PD-1/PD-L1 vs. Docetaxel/Paclitaxel). Subgroup analysis was performed based on the specific types of tumors in the experimental and control groups. **(A2)** Forest plots for the odds ratio of treatment related peripheral neuropathy (PD-1/PD-L1 vs. monotherapy). Subgroup analysis was performed based on the drug type (PD-1 or PD-L1) of the experimental group. **(B1)** Forest plots for the odds ratio of treatment related peripheral neuropathy (PD-1/PD-L1 vs. Chemotherapy). Subgroup analysis was performed based on the drug type (PD-1 or PD-L1) of the experimental group. **(B2)** Forest plots for the odds ratio of treatment related peripheral neuropathy (PD-1/PD-L1 vs. Chemotherapy). Subgroup analysis was performed based on the specific types of tumors in the experimental and control groups. **(C1)** Forest plots for the odds ratio of treatment related peripheral neuropathy (PD-1/PD-L1 + Chemotherapy vs. Chemotherapy). Subgroup analysis was performed based on the drug type (PD-1 or PD-L1) of the experimental group. **(C2)** Forest plots for the odds ratio of treatment related peripheral neuropathy (PD-1/PD-L1 + Chemotherapy vs. Chemotherapy). Subgroup analysis was performed based on the specific types of tumors in the experimental and control groups.

In Group A, the incidence risk of PD-1/PD-L1 subgroup was obvious lower than the control group [OR = 0.13, 95%CI:[0.04, 0.45], *I*^2^ = 0%, *Z* = 3.24 (*p* = 0.001); Figure 3A; (19, 23, 27–29)]. Different grouping methods for subgroup analysis were adopted for dealing with all the data, no statistically significant difference was found among them (Figures 3A1,A2). No heterogeneity was found in Group A (*I*^2^ = 0%). Similar to the results of OR, RR, and RD of Group A were displayed in Supplemental Figures 8A, 10A. The corresponding funnel plots were summarized in Supplemental Figures 9A, 11A.

When PD-1/PD-L1 drugs were compared with chemotherapy (Group B), the incidence risk of peripheral neuropathy limited to grade 3–5 was significantly lower than that of the control group, and the OR results are summarized in Figure 3B [OR = 0.11, 95%CI:[0.03, 0.49], *I*^2^ = 0%, Z = 2.92 (*P* = 0.004)] (20, 22, 30). The funnel plots of OR for Group B could be seen in Supplemental Figures 3B1,B2. Similar to the results of OR, RR, and RD of Group B were displayed in Supplemental Figures 8B, 10B.The corresponding funnel plots were gathered in Supplemental Figures 9B, 11B. No heterogeneity was found in Group B (*I*^2^ = 0%) (20, 22, 30).

The OR of Group C showed that the incidence risk of peripheral neuropathy in the PD-1/PD-L1 combined chemotherapy subgroup was significantly higher than that in the chemotherapy subgroup, and the *P*-value was statistically significant (Figure 3C) [OR = 1.74, 95%CI:[1.03, 2.92], *I*^2^ = 0%, *Z* = 2.09 (*P* = 0.04)] (15–18). The corresponding funnel plots of OR were gathered in Supplemental Figure 3C. No heterogeneity was found in Group C (*I*^2^ = 0%). Similar analysis results could also be seen in Supplemental Figure 8C, when the data of Group C was evaluated by RR [RR = 1.71, 95%CI:[1.03, 2.83], *I*^2^ = 0%, Z = 2.09 (*P* = 0.04)]. Different from OR and RR, the meta-analysis result was of no statistical significance (Supplemental Figure 10), when it was calculated by RD [RD = 0.01, 95%CI:[0.00, 0.02], I^2^ = 10%, Z = 1.82 (*P* = 0.07)]. The corresponding funnel plots of RD were gathered in Supplemental Figure 11C. Low heterogeneity related to RD was found in Group C (*I*^2^ = 10%). Subgroup analysis revealed that the source of heterogeneity might be related to the inclusion of this clinical trial (15).

## Discussion

Peripheral neuropathy is a painful condition deriving from many and varied etiologies (38, 39). Certain medications have been implicated in the iatrogenic development of drug induced peripheral neuropathy (DIPN) and include chemotherapeutic agents, antimicrobials, cardiovascular drugs, psychotropic, anticonvulsants, among others (39). Chemotherapy-induced peripheral neuropathy (CIPN), reported in several studies, especially for paclitaxel induced peripheral neuropathy, was common for cancer patients (40, 41). CIPN was a dose limiting toxicity, negatively impacting both quality of life and disease outcomes (42). However, during the process of anti-tumor treatment, combinations of drugs that were unknown to cause CIPN were prescribed for cancer patients, and sequential treatment for recurrence with additional CIPN-inducing drugs would also be suggested (43). Therefore, it would be difficult for us to determine which specific drug was responsible for the occurrence of peripheral neuropathy, especially for some newly marketed targeted anti-tumor drugs without fully understanding of toxicities, such as PD-1/PD-L1 inhibitors and Brentuximab vedotin (3, 14–30). To clarify the association between incidence risk of peripheral neuropathy and PD-1/PD-L1 inhibitors, we designed this meta-analysis.

After screening and eligibility assessment, a total of 17 clinical trials involving 10,500 patients were selected for the final meta-analysis. The flow diagram of the meta-analysis was shown in Figure 1, while the risk of bias summary was shown in Supplemental Figure 1. All clinical trials enrolled in the meta-analysis included at least one experimental group and one control group (14–30). Study quality and risk of bias among enrolled studies were checked by Newcastle-Ottawa scale (35). All clinical trials included were considered to be of higher quality. Therefore, the analytical conclusions based on the data of these clinical trials could represent certain reliability, authenticity, and credibility (14–30). In this study, we tried as many subgroup analysis methods as possible, and conducted a systematic and comprehensive analysis of the results, so the analysis results obtained were much more accurate (Figures 2, 3 and Supplemental Figures 4, 6, 8, 10) than that was analyzed just by one model.

The incidence of peripheral neuropathy for all grade was significantly lower in the PD-1/PD-L1 inhibitor group than that of the control group, either monotherapy (OR = 0.08, 95%CI:[0.03, 0.19], Figure 2A) or chemotherapy (OR = 0.05, 95%CI:[0.03, 0.11], Figure 2B) (14, 19–24, 26–30). Moderate heterogeneity was found in Group A (*I*^2^ = 69%) but Group B (*I*^2^ = 0%). Subgroup analysis results suggested that the source of heterogeneity was the PD-L1 subgroup (Figure 2A2) (21, 23, 26). The funnel plots of OR for Group A could be seen in Supplemental Figures 2A1,A2. Similar to the results of OR, Forest plots of RR and RD for Group A were displayed in Supplemental Figures 4A, 6A. The corresponding funnel plots were summarized in Supplemental Figures 5A, 7A. We found the existence of asymmetry of the funnel plot of Group A analysis (19, 21, 23, 26–29), so we concluded that there might be publication bias, but we could not rule out the possibility of asymmetry caused by other factors. Similar incidence risk of peripheral neuropathy for grade 3–5 could also be seen in Figure 3A (OR = 0.13, 95%CI:[0.04, 0.45]) (19, 23, 27–29). However, the heterogeneity (*I*^2^ = 0%) and the asymmetry of the funnel chart were not found [Supplemental Figure 3A; (19, 23, 27–29)]. Based on the above analysis results, we concluded that the heterogeneity and the asymmetry of the funnel plot were mainly derived from those two clinical trials (21, 26).

When PD-1/PD-L1 inhibitors were used in combination with chemotherapy (Group C), the risk of peripheral neuropathy was higher than in the control chemotherapy group, whether it was all-grade (OR = 1.22, 95%CI:[1.00, 1.49], Figure 2C) or grade 3–5 degree (OR = 1.74, 95%CI:[1.03, 2.92], Figure 3C) (15–18). Similar incidence trend could also be obtained when they were evaluated by RR (Supplemental Figures 4C, 8C). No obviously statistical significant results of RD were only seen in Supplemental Figures 6C, 8C. Obvious heterogeneity and the asymmetry of the funnel chart were not found in Group C (Supplemental Figures 2C, 3C, 5C, 7C, 9C, 11C). It proved that the analytical conclusions we had obtained were credible.

A lot of clinical trials had reported that PD-1/PD-L1 inhibitors had better safety and satisfactory clinical efficacy in the process of anti-tumor therapy (14–30, 44). In the experimental subgroup of Group A and Group B, using PD-1/PD-L1 inhibitors alone, the incidence rate of peripheral neuropathy for grade 3–5 was 0% (19, 20, 22, 23, 27–30). In other words, if we encounter peripheral neuropathy of grade 3–5 in the course of anti-tumor therapy, the possibility caused by the PD-1/PD-L1 inhibitor was firstly excluded. Chemotherapy-induced peripheral neuropathy (CIPN), reported in several studies, especially for paclitaxel induced peripheral neuropathy, was common for cancer patients (40, 41). Stopping the use of related drugs remained to be the primary principle for the treatment of drug-related peripheral neuropathy. However, stopping all anti-tumor treatment for cancer patients, especially for advanced cancer patients, might lead to rapid progression of the tumor, and even endanger the patient's life. Based on the results of our analysis, we found that PD-1/PD-L1 inhibitors often played a secondary role for patients suffering from severe drug-related peripheral neuropathy [Figures 2, 3; (14–30)]. Therefore, when it was necessary to stop anti-tumor therapy to alleviate severe peripheral neuropathy in patients, chemotherapy drugs other than PD-1/PD-L1 would be considered first (Figures 2C, 3C) (15–18). This finding had an important clinical guiding significance in clinical work.

## Conclusions

Compared with chemotherapy, incidence risk of peripheral neuropathy related to PD-1/PD-L1 inhibitor was significantly lower than that of the chemotherapy group, while PD-1/PD-L1 inhibitor increased the incidence risk of peripheral neuropathy when it was combined with chemotherapy.

## Ethics Statement

This study belongs to the type of data analysis and rearrangement, and does not involve human or animal related ethical issues.

## Author Contributions

YT had full access to all data in the study and all authors had final responsibility for the decision to submit for publication. ZS, SZ, XY, ND, and YT had the full data of the paper. MX, QZ, YL, LY, HS, JX, and YM were responsible for the collection of clinical data. ZS helped to gather online data and write the report.

### Conflict of Interest Statement

The authors declare that the research was conducted in the absence of any commercial or financial relationships that could be construed as a potential conflict of interest.

## Abbreviations

CI: confidence interval
CIPN: Chemotherapy-induced peripheral neuropathy
DIPN: drug induced peripheral neuropathy
FE: fixed effect
HR: hazard ratios
OR: odds ratio
PD-1: programmed cell death-1
PD-L1: programmed cell death ligand 1
PRISMA: Preferred Reporting Items for Systematic Reviews and Meta-Analyses
RD: risk difference
RE: random effect
RR: risk ratio.
