# Supplementary material for: The Association Between the Incidence Risk of Peripheral Neuropathy and PD-1/PD-L1 Inhibitors in the Treatment for Solid Tumor Patients: A Systematic Review and Meta-Analysis
Source: Front Oncol. 2019 Sep 4;9:866. doi: 10.3389/fonc.2019.00866 (PMC6736994; doi:10.3389/fonc.2019.00866)

**Supplemental Figure 1:**

**A:Risk of bias graph: review authors' judgements about each risk of bias item presented as percentages across all included studies.**

**B:Risk of bias summary: review authors' judgements about each risk of bias item for each included study.**


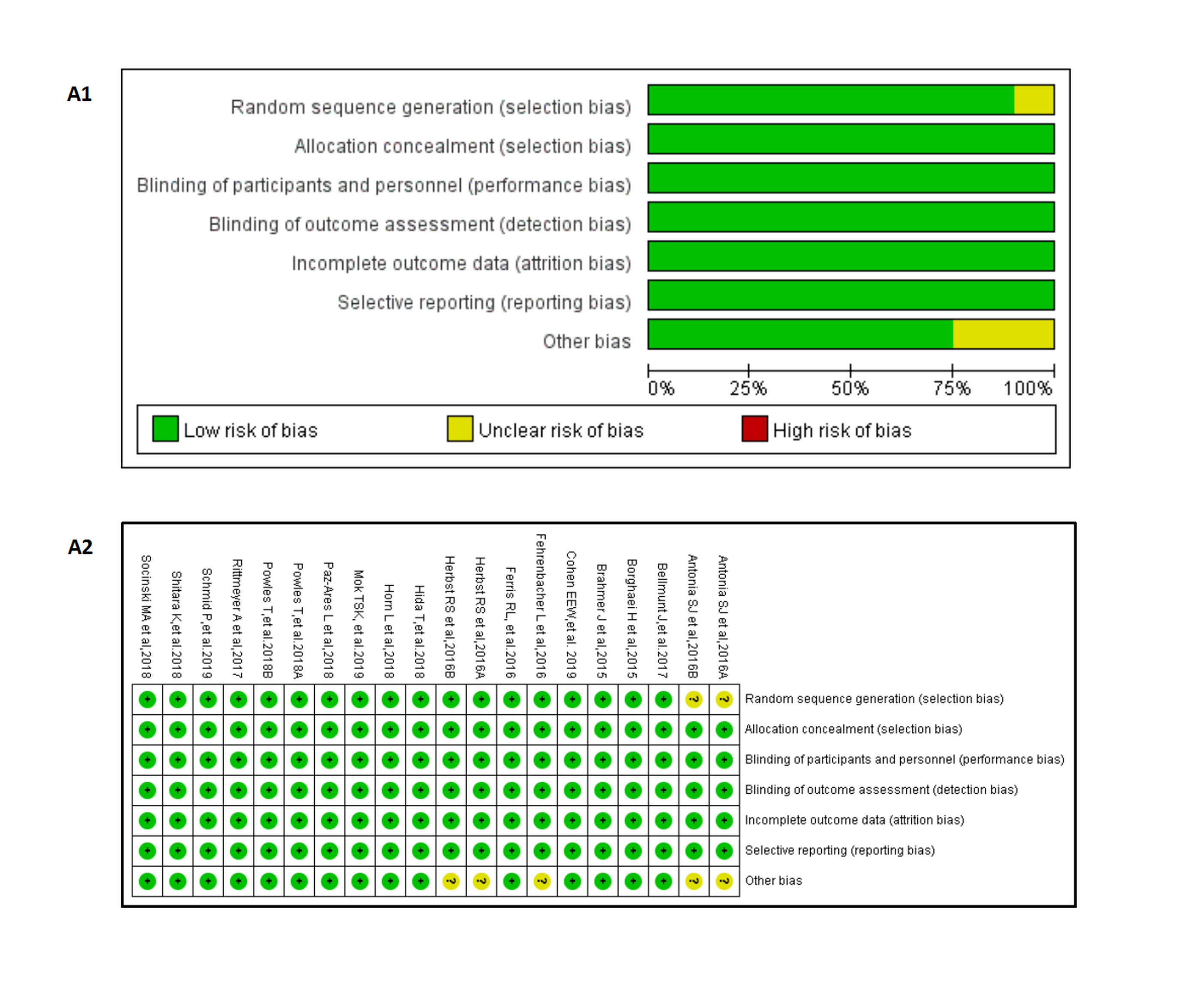


**Supplemental Figure 2: Funnel plots for the odds ratio of treatment related peripheral neuropathy for all grade.**

**A1:** Funnel plots for the odds ratio of treatment related peripheral neuropathy (PD-1/PD-L1 VS Docetaxel/Paclitaxel). Subgroup analysis was performed according to the type of chemotherapy drug in the control group.

**A2:** Funnel plots for the odds ratio of treatment related peripheral neuropathy (PD-1/PD-L1 VS monotherapy). Subgroup analysis was performed based on the drug type (PD-1 or PD-L1) of the experimental group.

**B1:** Funnel plots for the odds ratio of treatment related peripheral neuropathy (PD-1/PD-L1 VS Chemotherapy). Subgroup analysis was performed based on the drug type (PD-1 or PD-L1) of the experimental group.

**B2:** Funnel plots for the odds ratio of treatment related peripheral neuropathy (PD-1/PD-L1 VS Chemotherapy). Subgroup analysis was performed based on the specific types of tumors in the experimental and control groups.

**C1:** Funnel plots for the odds ratio of treatment related peripheral neuropathy (PD-1/PD-L1+ Chemotherapy VS Chemotherapy). Subgroup analysis was performed based on the drug type (PD-1 or PD-L1) of the experimental group.

**C2:** Funnel plots for the odds ratio of treatment related peripheral neuropathy (PD-1/PD-L1+ Chemotherapy VS Chemotherapy). Subgroup analysis was performed based on the specific types of tumors in the experimental and control groups.


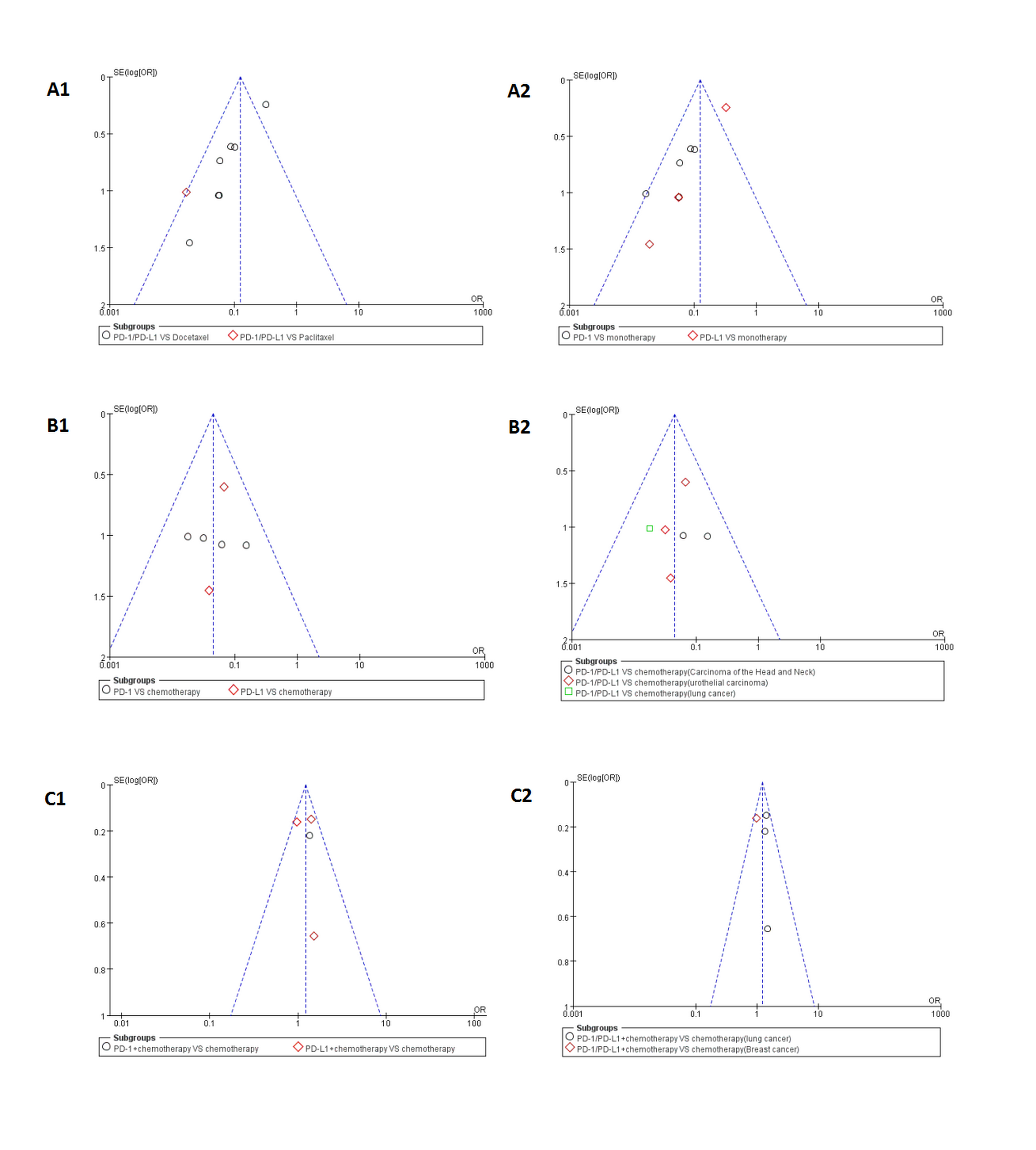


**Supplemental Figure 3：Funnel plots for the odds ratio of treatment related peripheral neuropathy for grade 3-5.**

**A1:** Funnel plots for the odds ratio of treatment related peripheral neuropathy (PD-1/PD-L1 VS Docetaxel/Paclitaxel). Subgroup analysis was performed based on the specific types of tumors in the experimental and control groups.

**A2:** Funnel plots for the odds ratio of treatment related peripheral neuropathy (PD-1/PD-L1 VS monotherapy). Subgroup analysis was performed based on the drug type (PD-1 or PD-L1) of the experimental group.

**B1:** Funnel plots for the odds ratio of treatment related peripheral neuropathy (PD-1/PD-L1 VS Chemotherapy). Subgroup analysis was performed based on the drug type (PD-1 or PD-L1) of the experimental group.

**B2:** Funnel plots for the odds ratio of treatment related peripheral neuropathy (PD-1/PD-L1 VS Chemotherapy). Subgroup analysis was performed based on the specific types of tumors in the experimental and control groups.

**C1:** Funnel plots for the odds ratio of treatment related peripheral neuropathy (PD-1/PD-L1+ Chemotherapy VS Chemotherapy). Subgroup analysis was performed based on the drug type (PD-1 or PD-L1) of the experimental group.

**C2:** Funnel plots for the odds ratio of treatment related peripheral neuropathy (PD-1/PD-L1+ Chemotherapy VS Chemotherapy). Subgroup analysis was performed based on the specific types of tumors in the experimental and control groups.


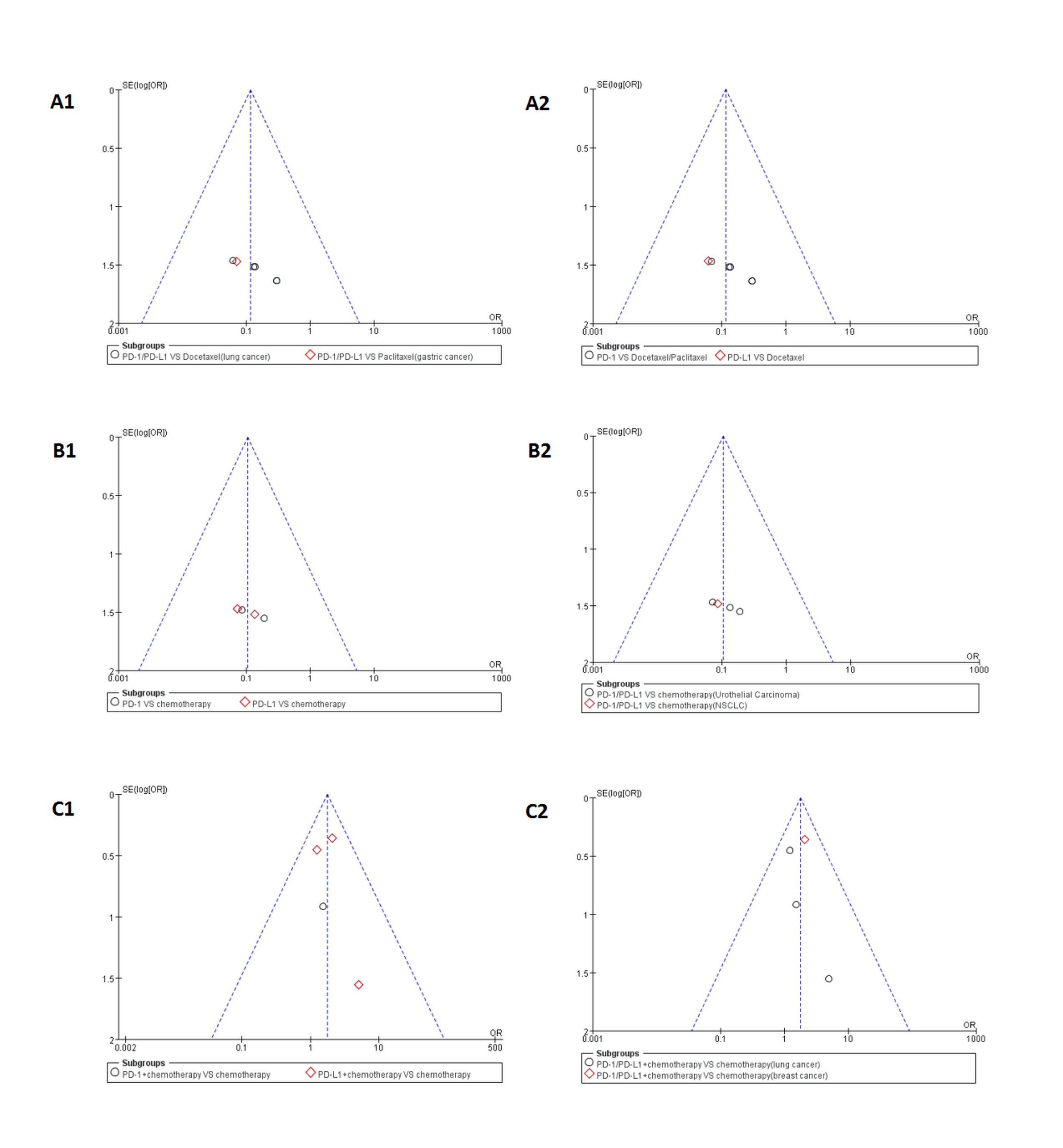


**Supplemental Figure 4: Forest plots for the risk ratio of treatment related peripheral neuropathy for all grade.**

**A1:** Forest plots for the risk ratio of treatment related peripheral neuropathy (PD-1/PD-L1 VS Docetaxel/Paclitaxel). Subgroup analysis was performed according to the type of chemotherapy drug in the control group.

**A2:** Forest plots for the risk ratio of treatment related peripheral neuropathy (PD-1/PD-L1 VS monotherapy). Subgroup analysis was performed based on the drug type (PD-1 or PD-L1) of the experimental group.

**B1:** Forest plots for the risk ratio of treatment related peripheral neuropathy (PD-1/PD-L1 VS Chemotherapy). Subgroup analysis was performed based on the drug type (PD-1 or PD-L1) of the experimental group.

**B2:** Forest plots for the risk ratio of treatment related peripheral neuropathy (PD-1/PD-L1 VS Chemotherapy). Subgroup analysis was performed based on the specific types of tumors in the experimental and control groups.

**C1:** Forest plots for the risk ratio of treatment related peripheral neuropathy (PD-1/PD-L1+ Chemotherapy VS Chemotherapy). Subgroup analysis was performed based on the drug type (PD-1 or PD-L1) of the experimental group.

**C2:** Forest plots for the risk ratio of treatment related peripheral neuropathy (PD-1/PD-L1+ Chemotherapy VS Chemotherapy). Subgroup analysis was performed based on the specific types of tumors in the experimental and control groups.


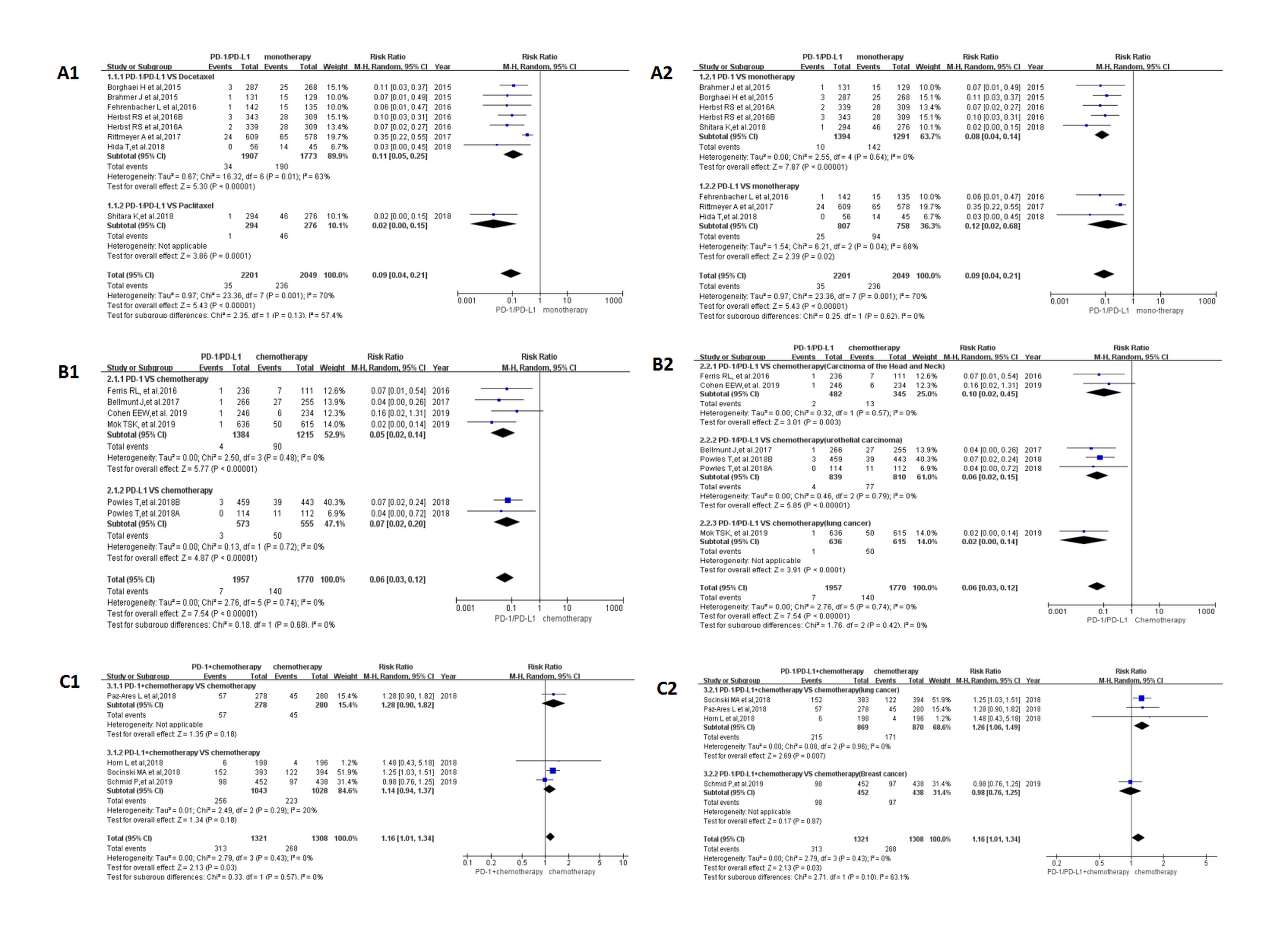


**Supplemental Figure 5: Funnel plots for the risk ratio of treatment related peripheral neuropathy for all grade.**

**A1:** Funnel plots for the risk ratio of treatment related peripheral neuropathy (PD-1/PD-L1 VS Docetaxel/Paclitaxel). Subgroup analysis was performed according to the type of chemotherapy drug in the control group.

**A2:** Funnel plots for the risk ratio of treatment related peripheral neuropathy (PD-1/PD-L1 VS monotherapy). Subgroup analysis was performed based on the drug type (PD-1 or PD-L1) of the experimental group.

**B1:** Funnel plots for the risk ratio of treatment related peripheral neuropathy (PD-1/PD-L1 VS Chemotherapy). Subgroup analysis was performed based on the drug type (PD-1 or PD-L1) of the experimental group.

**B2:** Funnel plots for the risk ratio of treatment related peripheral neuropathy (PD-1/PD-L1 VS Chemotherapy). Subgroup analysis was performed based on the specific types of tumors in the experimental and control groups.

**C1:** Funnel plots for the risk ratio of treatment related peripheral neuropathy (PD-1/PD-L1+ Chemotherapy VS Chemotherapy). Subgroup analysis was performed based on the drug type (PD-1 or PD-L1) of the experimental group.

**C2:** Funnel plots for the risk ratio of treatment related peripheral neuropathy (PD-1/PD-L1+ Chemotherapy VS Chemotherapy). Subgroup analysis was performed based on the specific types of tumors in the experimental and control groups.


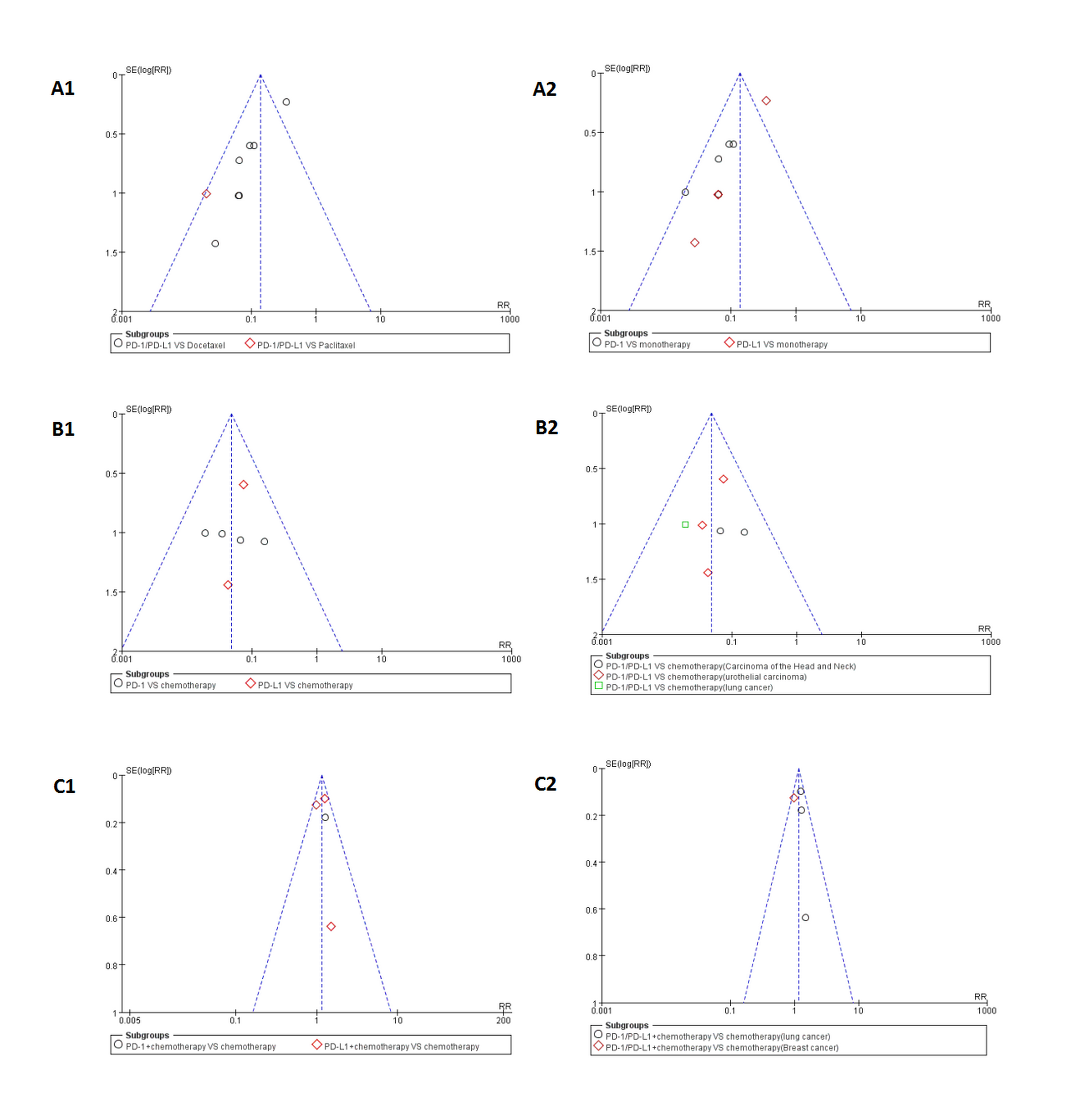


**Supplemental Figure 6: Forest plots for the risk difference of treatment related peripheral neuropathy for all grade.**

**A1:** Forest plots for the risk difference of treatment related peripheral neuropathy (PD-1/PD-L1 VS Docetaxel/Paclitaxel). Subgroup analysis was performed according to the type of chemotherapy drug in the control group.

**A2:** Forest plots for the risk difference of treatment related peripheral neuropathy (PD-1/PD-L1 VS monotherapy). Subgroup analysis was performed based on the drug type (PD-1 or PD-L1) of the experimental group.

**B1:** Forest plots for the risk difference of treatment related peripheral neuropathy (PD-1/PD-L1 VS Chemotherapy). Subgroup analysis was performed based on the drug type (PD-1 or PD-L1) of the experimental group.

**B2:** Forest plots for the risk difference of treatment related peripheral neuropathy (PD-1/PD-L1 VS Chemotherapy). Subgroup analysis was performed based on the specific types of tumors in the experimental and control groups.

**C1:** Forest plots for the risk difference of treatment related peripheral neuropathy (PD-1/PD-L1+ Chemotherapy VS Chemotherapy). Subgroup analysis was performed based on the drug type (PD-1 or PD-L1) of the experimental group.

**C2:** Forest plots for the risk difference of treatment related peripheral neuropathy (PD-1/PD-L1+ Chemotherapy VS Chemotherapy). Subgroup analysis was performed based on the specific types of tumors in the experimental and control groups.


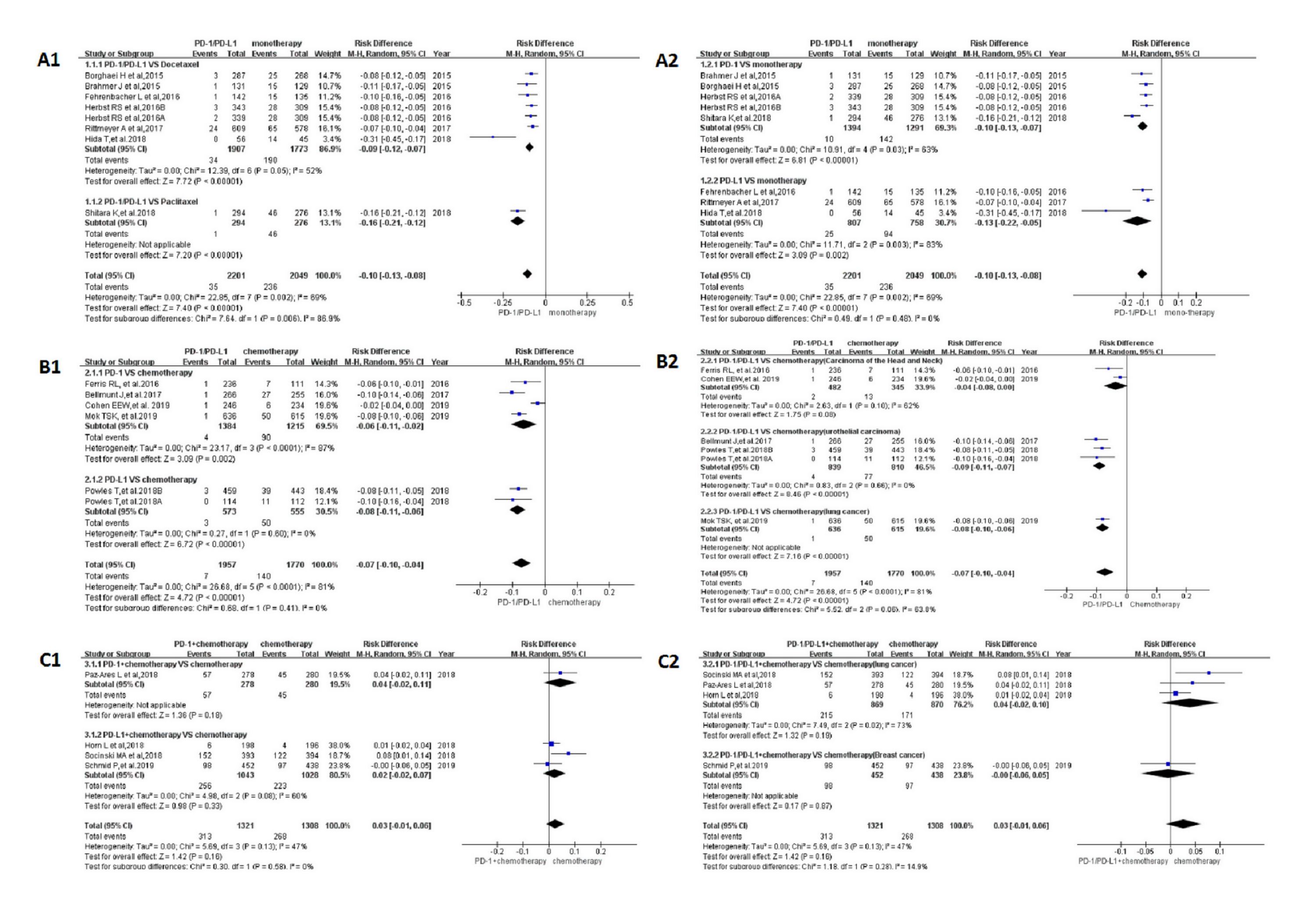


**Supplemental Figure 7: Funnel plots for the risk difference of treatment related peripheral neuropathy for all grade.**

**A1:** Funnel plots for the risk difference of treatment related peripheral neuropathy (PD-1/PD-L1 VS Docetaxel/Paclitaxel). Subgroup analysis was performed according to the type of chemotherapy drug in the control group.

**A2:** Funnel plots for the risk difference of treatment related peripheral neuropathy (PD-1/PD-L1 VS monotherapy). Subgroup analysis was performed based on the drug type (PD-1 or PD-L1) of the experimental group.

**B1:** Funnel plots for the risk difference of treatment related peripheral neuropathy (PD-1/PD-L1 VS Chemotherapy). Subgroup analysis was performed based on the drug type (PD-1 or PD-L1) of the experimental group.

**B2:** Funnel plots for the risk difference of treatment related peripheral neuropathy (PD-1/PD-L1 VS Chemotherapy). Subgroup analysis was performed based on the specific types of tumors in the experimental and control groups.

**C1:** Funnel plots for the risk difference of treatment related peripheral neuropathy (PD-1/PD-L1+ Chemotherapy VS Chemotherapy). Subgroup analysis was performed based on the drug type (PD-1 or PD-L1) of the experimental group.

**C2:** Funnel plots for the risk difference of treatment related peripheral neuropathy (PD-1/PD-L1+ Chemotherapy VS Chemotherapy). Subgroup analysis was performed based on the specific types of tumors in the experimental and control groups.


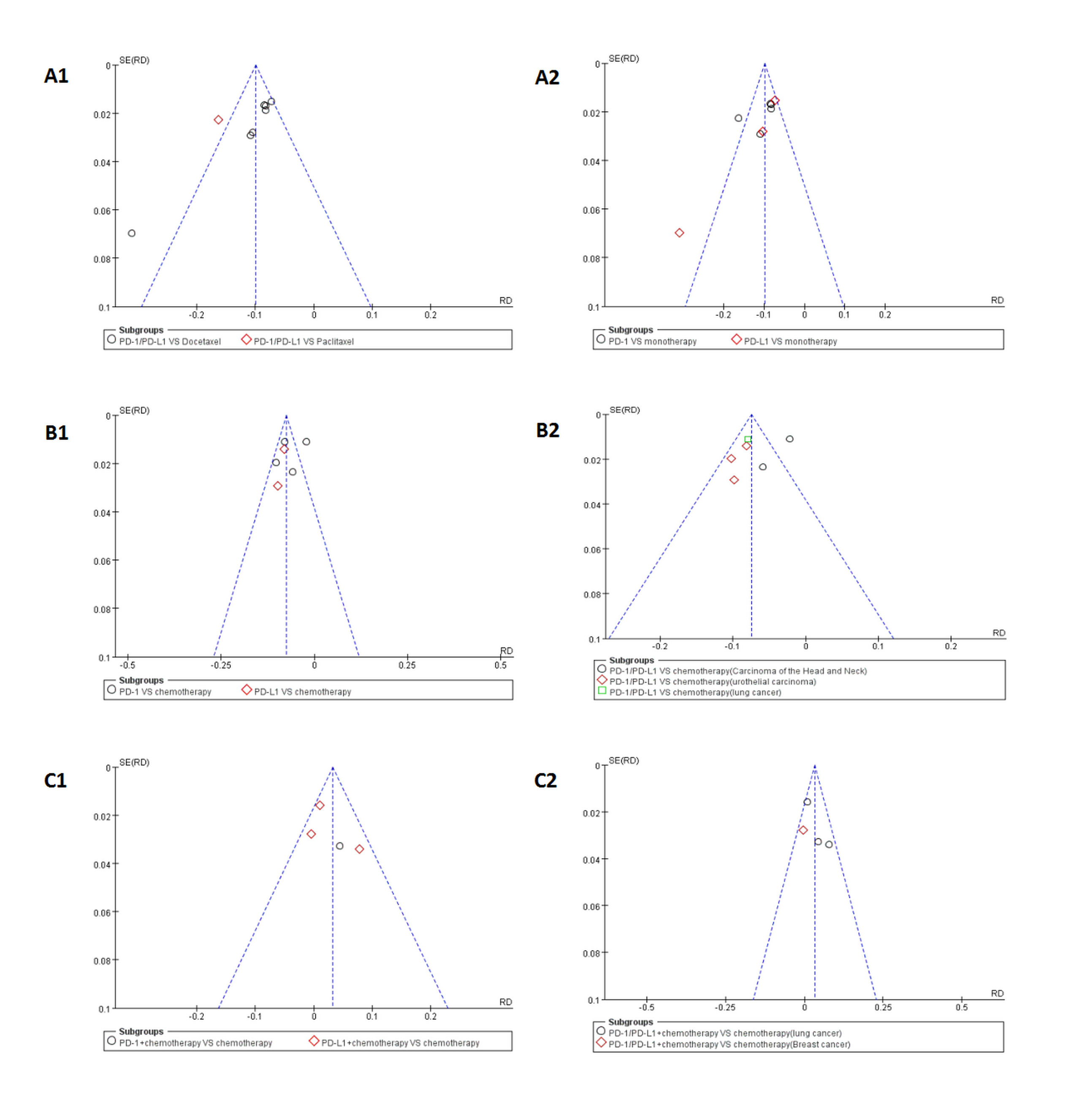


**Supplemental Figure 8：Forest plots for the risk ratio of treatment related peripheral neuropathy for grade 3-5.**

**A1:** Forest plots for the risk ratio of treatment related peripheral neuropathy (PD-1/PD-L1 VS Docetaxel/Paclitaxel). Subgroup analysis was performed based on the specific types of tumors in the experimental and control groups.

**A2:** Forest plots for the risk ratio of treatment related peripheral neuropathy (PD-1/PD-L1 VS monotherapy). Subgroup analysis was performed based on the drug type (PD-1 or PD-L1) of the experimental group.

**B1:** Forest plots for the risk ratio of treatment related peripheral neuropathy (PD-1/PD-L1 VS Chemotherapy). Subgroup analysis was performed based on the drug type (PD-1 or PD-L1) of the experimental group.

**B2:** Forest plots for the risk ratio of treatment related peripheral neuropathy (PD-1/PD-L1 VS Chemotherapy). Subgroup analysis was performed based on the specific types of tumors in the experimental and control groups.

**C1:** Forest plots for the risk ratio of treatment related peripheral neuropathy (PD-1/PD-L1+ Chemotherapy VS Chemotherapy). Subgroup analysis was performed based on the drug type (PD-1 or PD-L1) of the experimental group.

**C2:** Forest plots for the risk ratio of treatment related peripheral neuropathy (PD-1/PD-L1+ Chemotherapy VS Chemotherapy). Subgroup analysis was performed based on the specific types of tumors in the experimental and control groups.


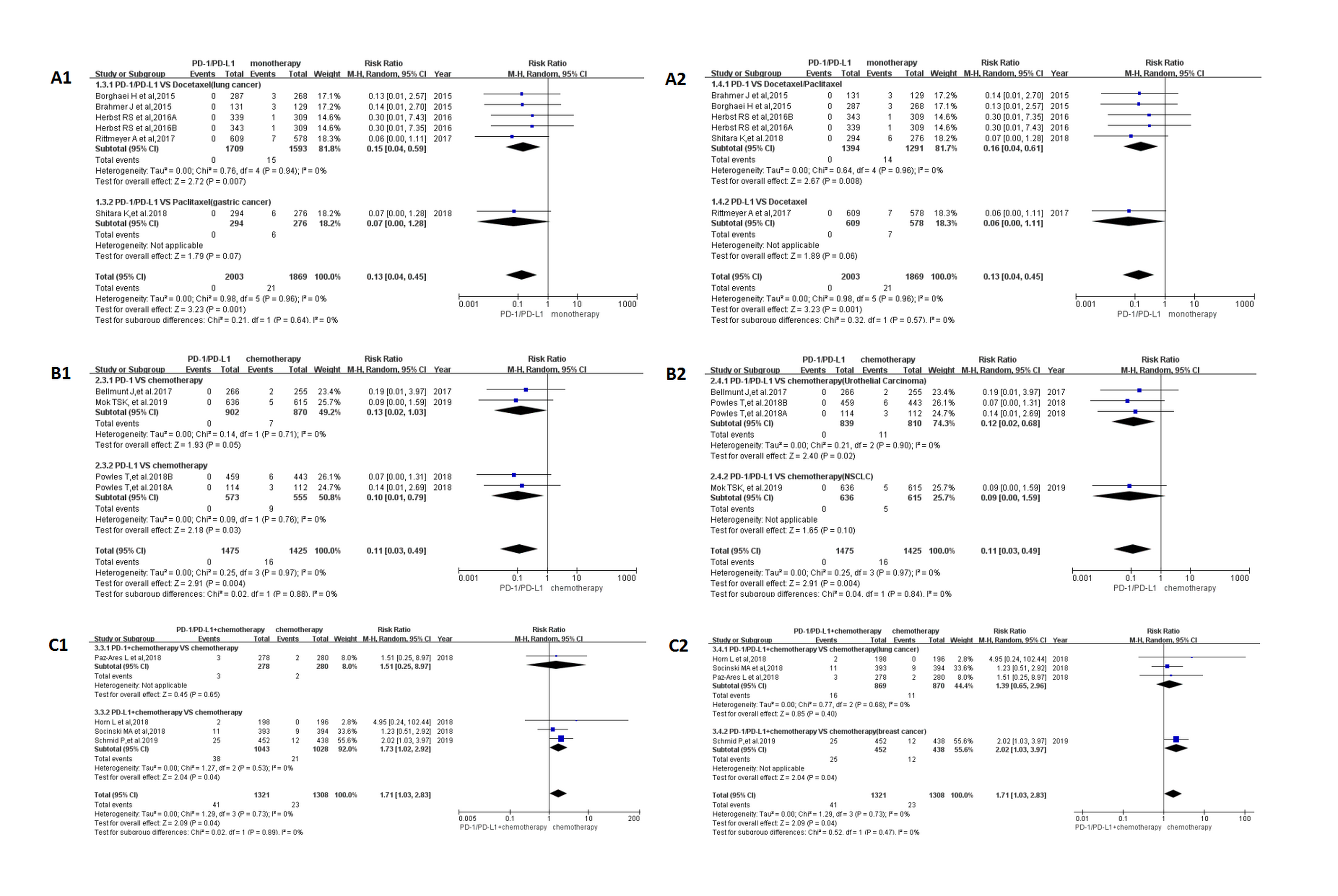


**Supplemental Figure 9：Funnel plots for the risk ratio of treatment related peripheral neuropathy for grade 3-5.**

**A1:** Funnel plots for the risk ratio of treatment related peripheral neuropathy (PD-1/PD-L1 VS Docetaxel/Paclitaxel). Subgroup analysis was performed based on the specific types of tumors in the experimental and control groups.

**A2:** Funnel plots for the risk ratio of treatment related peripheral neuropathy (PD-1/PD-L1 VS monotherapy). Subgroup analysis was performed based on the drug type (PD-1 or PD-L1) of the experimental group.

**B1:** Funnel plots for the risk ratio of treatment related peripheral neuropathy (PD-1/PD-L1 VS Chemotherapy). Subgroup analysis was performed based on the drug type (PD-1 or PD-L1) of the experimental group.

**B2:** Funnel plots for the risk ratio of treatment related peripheral neuropathy (PD-1/PD-L1 VS Chemotherapy). Subgroup analysis was performed based on the specific types of tumors in the experimental and control groups.

**C1:** Funnel plots for the risk ratio of treatment related peripheral neuropathy (PD-1/PD-L1+ Chemotherapy VS Chemotherapy). Subgroup analysis was performed based on the drug type (PD-1 or PD-L1) of the experimental group.

**C2:** Funnel plots for the risk ratio of treatment related peripheral neuropathy (PD-1/PD-L1+ Chemotherapy VS Chemotherapy). Subgroup analysis was performed based on the specific types of tumors in the experimental and control groups.


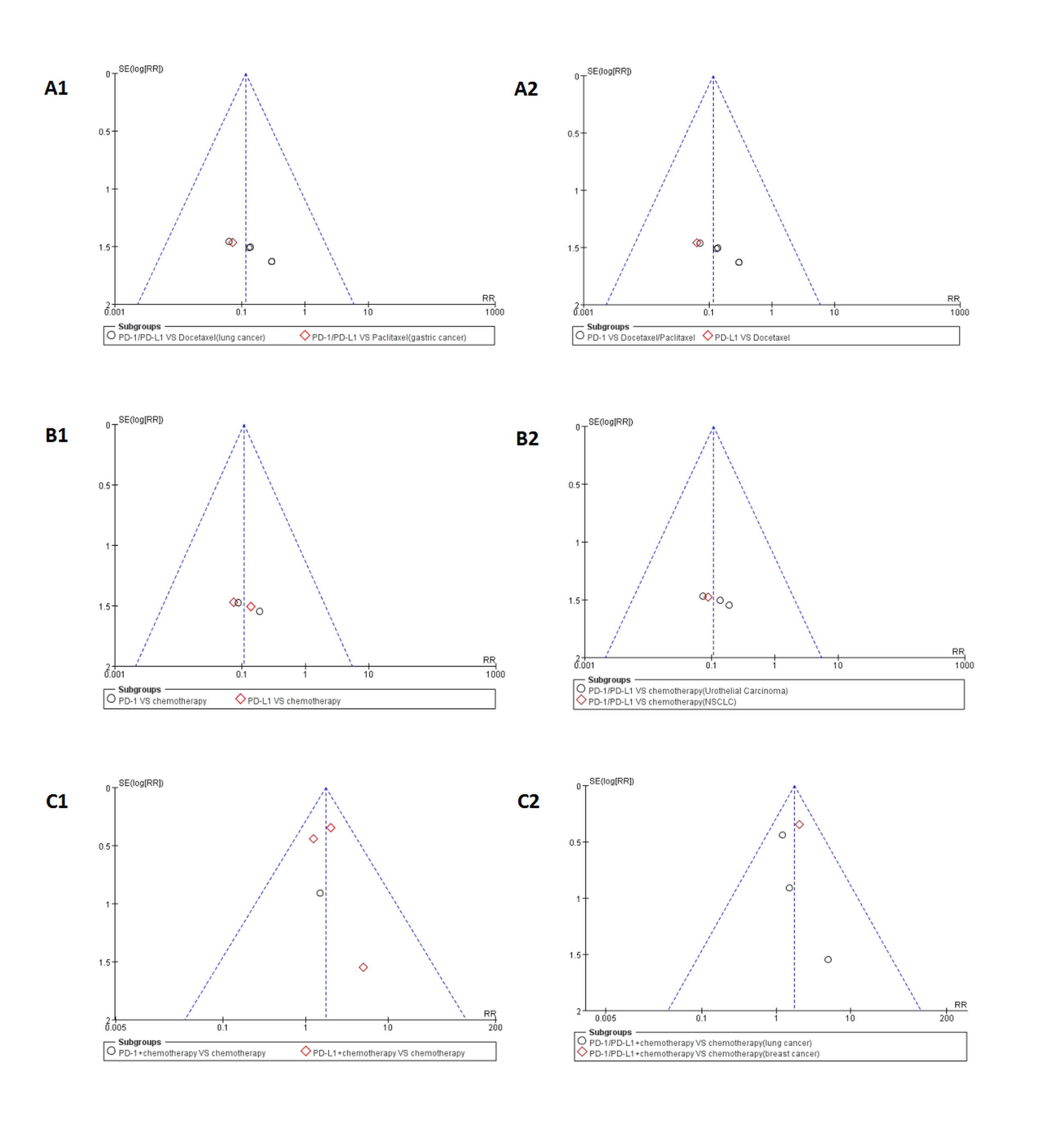


**Supplemental Figure 10：Forest plots for the risk difference of treatment related peripheral neuropathy for grade 3-5.**

**A1:** Forest plots for the risk difference of treatment related peripheral neuropathy (PD-1/PD-L1 VS Docetaxel/Paclitaxel). Subgroup analysis was performed based on the specific types of tumors in the experimental and control groups.

**A2:** Forest plots for the risk difference of treatment related peripheral neuropathy (PD-1/PD-L1 VS monotherapy). Subgroup analysis was performed based on the drug type (PD-1 or PD-L1) of the experimental group.

**B1:** Forest plots for the risk difference of treatment related peripheral neuropathy (PD-1/PD-L1 VS Chemotherapy). Subgroup analysis was performed based on the drug type (PD-1 or PD-L1) of the experimental group.

**B2:** Forest plots for the risk difference of treatment related peripheral neuropathy (PD-1/PD-L1 VS Chemotherapy). Subgroup analysis was performed based on the specific types of tumors in the experimental and control groups.

**C1:** Forest plots for the risk difference of treatment related peripheral neuropathy (PD-1/PD-L1+ Chemotherapy VS Chemotherapy). Subgroup analysis was performed based on the drug type (PD-1 or PD-L1) of the experimental group.

**C2:** Forest plots for the risk difference of treatment related peripheral neuropathy (PD-1/PD-L1+ Chemotherapy VS Chemotherapy). Subgroup analysis was performed based on the specific types of tumors in the experimental and control groups.


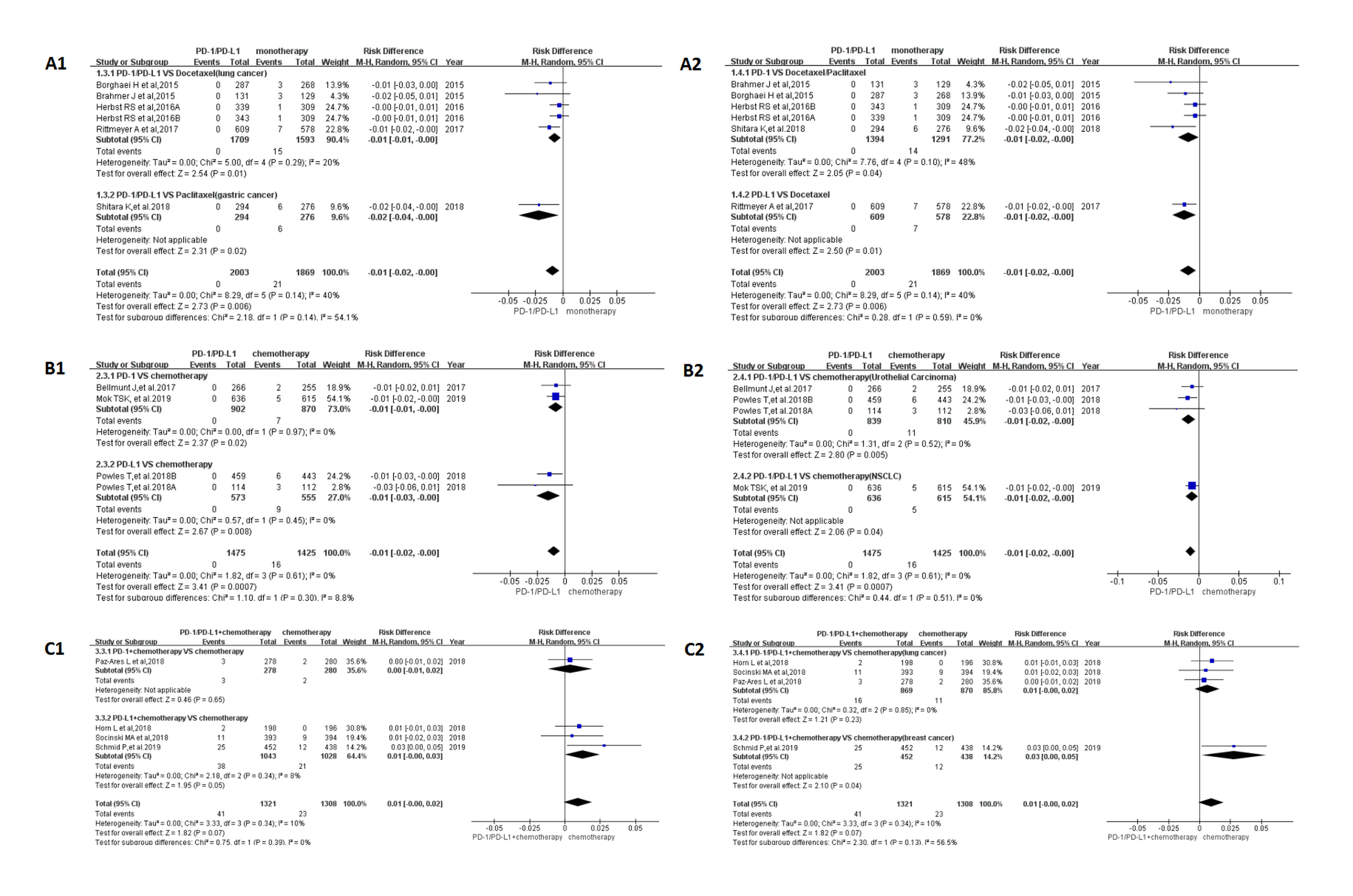


**Supplemental Figure 11：Funnel plots for the risk difference of treatment related peripheral neuropathy for grade 3-5.**

**A1:** Funnel plots for the risk difference of treatment related peripheral neuropathy (PD-1/PD-L1 VS Docetaxel/Paclitaxel). Subgroup analysis was performed based on the specific types of tumors in the experimental and control groups.

**A2:** Funnel plots for the risk difference of treatment related peripheral neuropathy (PD-1/PD-L1 VS monotherapy). Subgroup analysis was performed based on the drug type (PD-1 or PD-L1) of the experimental group.

**B1:** Funnel plots for the risk difference of treatment related peripheral neuropathy (PD-1/PD-L1 VS Chemotherapy). Subgroup analysis was performed based on the drug type (PD-1 or PD-L1) of the experimental group.

**B2:** Funnel plots for the risk difference of treatment related peripheral neuropathy (PD-1/PD-L1 VS Chemotherapy). Subgroup analysis was performed based on the specific types of tumors in the experimental and control groups.

**C1:** Funnel plots for the risk difference of treatment related peripheral neuropathy (PD-1/PD-L1+ Chemotherapy VS Chemotherapy). Subgroup analysis was performed based on the drug type (PD-1 or PD-L1) of the experimental group.

**C2:** Funnel plots for the risk difference of treatment related peripheral neuropathy (PD-1/PD-L1+ Chemotherapy VS Chemotherapy). Subgroup analysis was performed based on the specific types of tumors in the experimental and control groups.


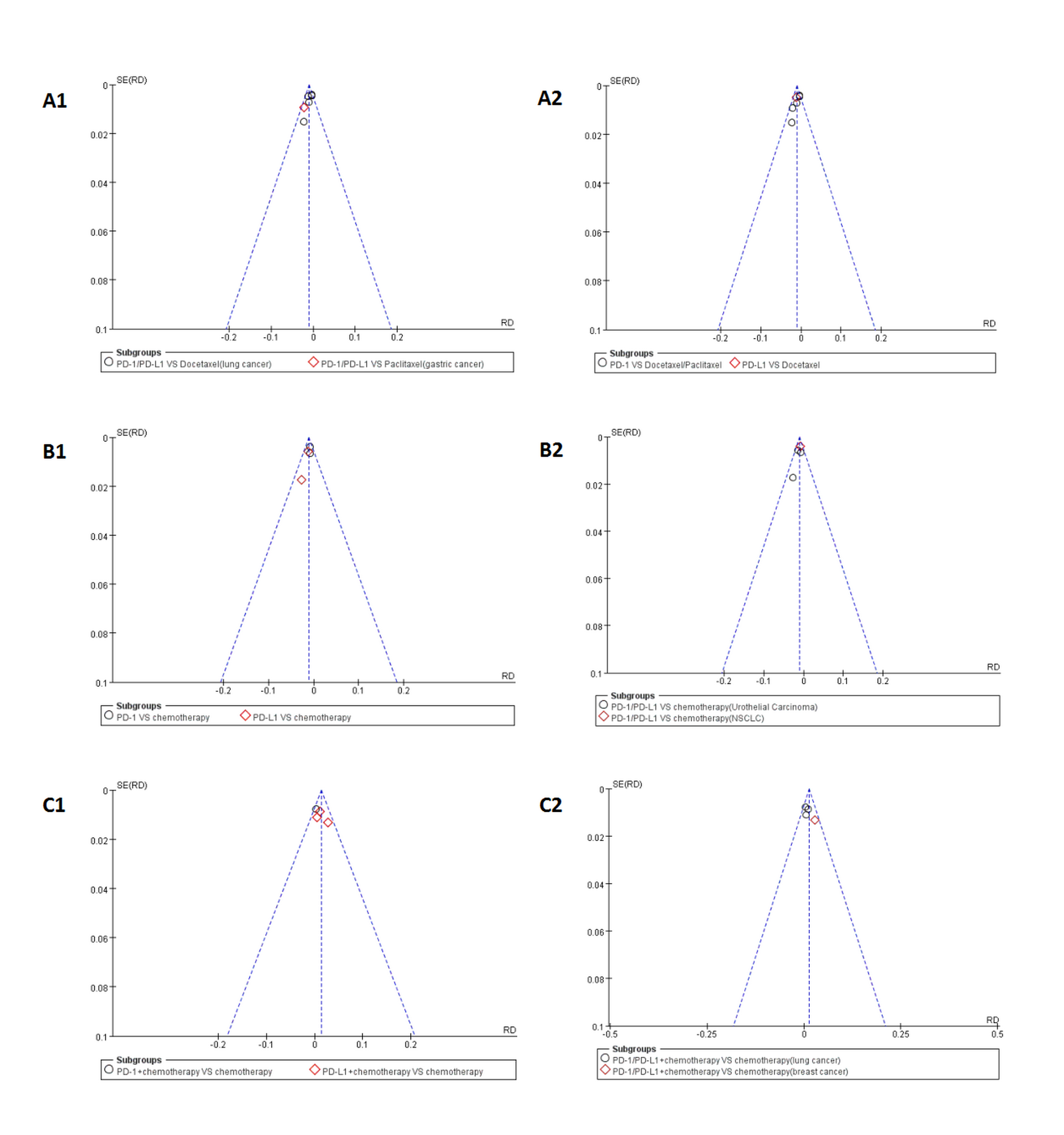

Supplement: Supplementary file 1 [file Data_Sheet_1.doc]
